# Characteristics and clinical outcome of patients with hypereosinophilia of undetermined significance

**DOI:** 10.1007/s12032-013-0815-1

**Published:** 2013-12-14

**Authors:** Grzegorz Helbig, Marek Hus, Tomasz Francuz, Joanna Dziaczkowska-Suszek, Anna Soja, Sławomira Kyrcz-Krzemień

**Affiliations:** 1 Department of Hematology and Bone Marrow Transplantation, Silesian Medical University, Dabrowski Street 25, 40-032 Katowice, Poland; 2 Department of Hematology, Medical University of Lublin, Lublin, Poland; 3 Department of Biochemistry, Silesian Medical University, Katowice, Poland

**Keywords:** Hypereosinophilia of undetermined significance, Hypereosinophilic syndrome, Corticosteroids, Outcome

## Abstract

The term “hypereosinophilia of undetermined significance” (HE_us_) previously known as idiopathic, benign eosinophilia relates to patients who have a long-lasting, unexplained and asymptomatic blood HE. These patients have not been studied so far in terms of demographic characteristics and clinical outcome. The aim of this study was to present the clinical characteristics and outcome of HE_us_ patients. This is a retrospective, single-center study of 40 patients with HE_us_. All patients underwent the basic and specialized evaluations in order to rule out the most common causes of blood HE, but no abnormalities were detected. Twelve patients with at least moderate blood hypereosinophilia (defined as greater than 3.0 × 10^9^/L) for more than 1-year duration were treated with corticosteroids (CS) to avoid end-organ damage. Twenty-one patients (52 %) had an increased leukocyte count at diagnosis. Median blood eosinophilia was 4.2 × 10^9^/L (range 1.5–55.4). HE > 3.0 × 10^9^/L was demonstrated in 17 patients. 65 % of studied population had an increased serum IgE levels, whereas only 2 % demonstrated an increased serum vitamin B12 levels. A median bone marrow infiltration by eosinophils was 30.5 % (range 11–78.2). All treated patients responded promptly to CS and remained in complete remission while receiving low doses of CS (20 mg/day to 5 mg every 2-day). One patient developed hypereosinophilic syndrome (HES) after 11 years of follow-up. Further studies are needed to define risk factors of HES development. The use of CS for HE_us_ patients is controversial and should be individualized.

## Introduction

Blood hypereosinophilia (HE) remains a frequent finding encountered in a daily clinical practice of different fields of medicine. Under various conditions, eosinophils may produce and release a variety of biologically active substances which may invade target organ and lead to its dysfunction and/or damage. The harmful role of eosinophils results from their inflammatory, fibrotic and thrombotic properties [1]. The production and development of eosinophils are regulated by several cytokines, but the role of interleukin (IL)-5 was found to be essential. It is secreted by activated T cells and to a lesser extent by mast cells and eosinophils themselves [2].

The underlying causes of HE are diverse and can be broadly divided into primary (clonal), secondary (reactive), hereditary (familial) and idiopathic. The term HE should be used when blood eosinophilia is greater than 1.5 × 10^9^/L on two occasions with a minimum of 1-month time interval, and/or tissue HE is documented. The contemporary definition of hypereosinophilic syndrome (HES) requires the presence of blood HE and an end-organ damage that was proved to be eosinophilia related [3].

As the characteristics of different HES variants are well-known [4], the outcome of patients with asymptomatic, unexplained and persistent HE remains unclear. Herein, we present the clinical and laboratory characteristics of 40 consecutive patients with idiopathic blood HE.

## Materials and methods

All patients included in the study met the criteria for blood HE, and they were recruited from several hematologic centers in Poland between 1994 and 2013. The reasons why the patients visited the primary care physician and performed full blood test were following: random blood investigation (*n* = 30), abdominal pain (*n* = 2), loss of weight (*n* = 2), facial swelling (*n* = 1), joint pain (*n* = 1), bone pain (*n* = 1), night sweats (*n* = 1), diarrhea (*n* = 1) and dyspnoea (*n* = 1). All patients underwent the basic evaluations at the primary care level including all necessary tests in order to rule out the most common causes of blood HE, but no abnormalities were detected. Physical examination was normal as well as chest X-ray and abdominal ultrasound. Originally reported complaints resolved spontaneously after 2–3 days without treatment, and they were found not to be HE related. Therefore, more detailed imaging and endoscopic studies were not recommended by a treating physician. Moreover, these symptoms did not re-appear in the long-term observation.

Due to the persistent blood HE, these patients were referred to a hematologist. All patients were free of any symptoms. On admission a complete blood test with differential, biochemistry and urinalysis were repeated. After the blood HE has been confirmed, the more specialized studies were initiated. Serum vitamin B12 and immunoglobulin E (IgE) levels were measured. The presence of infections caused by human immunodeficiency virus, cytomegalovirus, Epstein-Barr virus, hepatitis B virus, hepatitis C virus and toxocara was excluded using serological tests. Anti-nuclear antibodies as well as myeloperoxidase and proteinase 3 anti-neutrophil cytoplasmic antibodies were ruled out. Every patient had negative echocardiography and pulmonary function tests. No patients had indications for computed tomography or magnetic resonance imaging studies. Peripheral blood T-lymphocytes were determined by flow cytometry technique on EPICS-XL-MCL (Beckman-Coulter, USA) using monoclonal antibodies directed against T-cell surface antigens: CD2, CD3, CD4, CD5, CD7, CD8, T-cell receptor (TCR) αβ and TCRγδ, and no aberrant population (including the most common CD3-CD4+) has been detected. Polymerase chain reaction (PCR) was negative for *JAK2*V617F, *BCR*-*ABL* and *FIP1L1*-*PDGFRA* molecular abnormalities. Bone marrow cytology was normal except eosinophilia. Bone marrow histology was not done. Blood tests with differential, biochemistry, electrocardiogram, echocardiography, chest X-ray and abdominal ultrasound were repeated every 3 months. Stool examination for ova and larvae was systematically repeated throughout the study period, and it remained negative. The study was approved by Local Ethics Committee by Silesian Medical University, Katowice, Poland. Two patients from the current study have been reported elsewhere [5].

## Treatment and response

Twelve patients with at least moderate blood eosinophilia (defined as greater than 3.0 × 10^9^/L) for more than 1-year duration were treated with corticosteroids to avoid end-organ damage. The starting CS dose varied between patients, and it was left to physician’s discretion. A complete response (CR) was defined as a return of absolute eosinophil count (AEC) to normal ranges (<0.7 × 10^9^/L). A maintenance dose was defined as the minimal effective CS dose needed for CR maintenance. The response to CS was assessed daily during the first week, then weekly within the first month, and monthly thereafter.

## Results

Forty patients (28 females and 12 males) at a median age at diagnosis of 61 years (range 17–85) were included in this retrospective study. A distant history of allergic skin reactions and helminth infections was present for five and four patients, respectively. Before entering the study, all patients received tinidazole to prevent giardiasis with no effect on blood eosinophilia. Twenty-one patients (52 %) had an increased white blood cell (WBC) count at diagnosis. Hemoglobin concentration <12 g/dL and platelet count <150 × 10^9^/L were reported for nine and two patients, respectively. These abnormalities normalized spontaneously and were found not to be eosinophilia related. Median AEC was 4.2 × 10^9^/L (range 1.5–55.4). An AEC greater than 3.0 × 10^9^/L was demonstrated for 17 (40 %) patients. 65 % of studied patients had an increased serum IgE levels, whereas only 2 % demonstrated an increased serum vitamin B12 levels. A median bone marrow infiltration by eosinophils was 30.5 % (range 11–78.2). No patient had an aberrant T-cell population on flow cytometry, however, T helper (Th)/T suppressor (Ts) ratio was found to be abnormal in eight patients. This ratio was increased in three patients and decreased in five. Cytogenetic studies revealed normal diploid karyotype in 30 patients, whereas no metaphases were obtained for the remaining 10. The median follow-up of the studied population was 55.2 months (range 6.4–231.4). Patients’ characteristics are shown in Table 1.

**Table 1:** Study group characteristics

Parameter	HE^US^ (*n*)
Number of patients	40
Gender: male/female	12/28
Median age (range; years)	61 (17–85)
WBC^a^ count (×10^9^/L)	11.2 (5.5–70.1)
WBC > 10 (×10^9^/L)	52 %
Hemoglobin (g/dL)	13.0 (8.7–19.1)
Hemoglobin <12 (g/dL)	22 %
Platelet count (×10^9^/L)	289 (68–605)
Platelet count <150 (×10^9^/L)	2 %
AEC^b^ (×10^9^/L)	4.2 (1.5–55.4)
AEC >3 × 10^9^/L	42 %
Eosinophils in bone marrow (%)	30.5 (11–78.2)
Serum IgE (IU/mL)^c^	528 (11.9–4,089)
Serum IgE > N^c^	67 %
Serum B12 vitamin (pg/mL)^c^	333 (149–1,431)
Serum B12 > N^c^	2 %

^a^ *WBC* white blood cell, ^b^ *AEC* absolute eosinophil count, ^c^ normal ranges (N): IgE < 100 IU/mL; for vitamin B12 level: 157–1,057 pg/mL

Twelve out of the 17 patients with at least moderate blood eosinophilia started the treatment with steroids whereas the remaining five refused the therapy. The starting CS dose varied between 5 and 60 mg daily, and the highest doses were reserved for patients with severe blood HE. The maintenance dose was fixed to maintain CR. All patients responded promptly to CS and remained in CR while receiving low doses of CS (20 mg/day to 5 mg every 2-day). The attempts of treatment discontinuation while in CR failed, and the patients were left on a minimal effective CS dose. No patient, who originally refused the therapy, received the follow-up treatment. Side effects of CS therapy were mild and included bone and joint pain and malaise. None of the patients needed CS dose reduction or discontinuation due to adverse events. Twenty-seven patients who remained off therapy had a stable blood HE, and no organ dysfunction was demonstrated during follow-up. One female with no prior CS developed eosinophilia-related cardiac failure after 11 years of sustained blood HE, and she was successfully treated with CS. Details of CS therapy are presented in Table 2.

**Table 2:** Steroids for hypereosinophilia of undetermined significance

No	AEC (x10^9^/L) at CS initiation	Serum IgE (IU/mL) at CS initiation	CS initial dose (mg/day)	CS maintenance dose (mg/day)	Time to CR in days
1.	3.73	11.9	15	5 every 2-day	<7
2.	4.25	1,143.0	20	10	<7
3.	3.55	1,033.0	10	10/5 alternately	14
4.	31.1	19.4	60	20	14
5.	3.1	528.0	20	5	<7
6.	3.8	2,550.0	10	5	<7
7.	4.65	43.0	5	5	14
8.	8.5	1,080.0	30	10	<7
9.	7.9	4,089.0	30	10	<7
10.	21.4	3,648.0	30	10	<7
11.	15.8	176.0	40	10	14
12.	55.4	361.0	60	20	14

*AEC* absolute eosinophil count, *CS* corticosteroids, *CR* complete response

## Discussion

The term “hypereosinophilia of undetermined significance” (HE_us_) previously known as idiopathic, benign eosinophilia, relates to patients who have a long-lasting, unexplained and asymptomatic blood HE. The pathogenesis and prognosis of such cases remain unknown, and its clinical implication is to be validated. By definition, HE_us_ patients have no reactive and malignant causes of blood HE. A family history remains also non-informative. Moreover, these cases do not develop an eosinophilia-related organ dysfunction or damage [3]. If so, they should be classified as HES. There are lacking prospective studies involving this patient population, especially in regard to clinical and laboratory characteristics as well as long-term follow-up. The risk of HES development in a long-dated observation remains unknown. There is also no consensus regarding CS introduction for these patients. Bearing all these issues in mind, this retrospective analysis was done.

Only single retrospective studies on a natural history of blood HE have been published so far [6, 7]. In fact, most reported patients had well-defined causes of HE, and only a small minority of cases was actually idiopathic. Thus, regarding these two large reports, we focus on the patients with idiopathic blood HE. Unfortunately, both reports had many drawbacks resulting from their retrospective nature. A recently published report [6] recruited 33 patients with presumably HE_us_, but in fact, the appropriate diagnostic work-up was performed in a minority of them, and it was restricted to some common investigations. Nevertheless, the presumably HE_us_ patients had AEC greater than 5.0 × 10^9^/L and an increased serum IgE levels. Most patients were treated with steroids and achieved a long-term response, but the doses of CS were not provided. Surprisingly, the median time to response to CS exceeded 2 months.

The other retrospective study included 100 hospitalized patients with blood HE, and 34 % of them had an unknown etiology despite an extensive panel of investigations [7]. This figure has also been demonstrated by others [8, 9]. Conversely, only 3 % of the patients had unexplained HE in a large study reported by Lombardi et al. [10]. However, one should keep in mind that all these studies were based on a chart review and they had many limitations resulting from its retrospective nature. Moreover, they did not provide the results of molecular studies (e.g., *FIP1L1*-*PDGFRA*). There was also lacking data on the clinical outcome in a long-term follow-up. Nevertheless, the vast majority of detected blood eosinophilias was associated with allergic processes [7, 8]. Kobayashi et al. [11] defined the threshold of blood HE that may indicate the presence of HES and distinguished it from bronchial asthma. It was demonstrated that AEC greater than 2.052 × 10^9^/L was strongly suggestive of HES, and this value was associated with a higher risk of organ damage. These patients had also high serum IgE levels. However, one should keep in mind that the definition of HES provided by authors differed significantly from that used nowadays. In a very recent paper, Chen et al. [12] reported on eight patients who met the current HE_us_ criteria [3]. When they compared the laboratory data between HE_us_ and untreated HES subjects, they found no difference except for a higher serum IgE and IL-13 levels in the latter group. Surprisingly, 50 % of HE_us_ patients had an aberrant T-cell population, but none of them developed clinical symptoms or organ damage [12].

The risk factors of HES development have not been established so far. Only one patient from our study cohort developed HES, and it has been 11 years since blood HE was detected. That was female with AEC varied between 1.5 × 10^9^–2.5 × 10^9^/L during the whole follow-up, with a slightly increased serum IgE level (146 IU/mL; normal range <100 IU/mL) and an abnormal Th/Ts ratio (0.7) at HE_us_ diagnosis. At the time of HES development, this case was deeper evaluated. Namely, a study for T-cell receptor clonal rearrangement by PCR was performed, and it was found to be positive. Serum thymus and activation-regulated chemokine and interleukin (IL)-5 levels using commercially available ELISA kits were also measured, and they were following: 178.7 pg/mL (sensitivity >7 pg/mL) and 6.6 (sensitivity >1.08 pg/mL), respectively. Taken together, the diagnosis of lymphocytic-HES was established.

Despite the fact that blood AEC was greater than 3.0 × 10^9^/L in more than 40 % of study patients and serum IgE levels exceeded normal ranges in almost 70 % of them, the organ involvements were not detected in subsequent imaging studies. However, the median duration of follow-up for our patient population is less than 5 years. Older reports demonstrated that HES may develop after 8–9 years of sustained blood HE [13]. Currently, it is difficult to support the thesis that a benign idiopathic blood HE is a smoldering form of HES. To confirm this hypothesis, the markers of eosinophil activity were measured in a patient with a long-standing blood HE. The test showed an impaired function of eosinophils, but it did not explain why and when they become deleterious to the organs [14].

Another interesting aspect relates to the early drug therapy for asymptomatic patients with HE_us_. There is no evidence that the introduction of CS, despite the lack of symptoms, will influence the natural history of these patients and prevent the HES development. It should be highlighted that the treatment with CS for HE_us_ remains controversial, and currently, there is no data supporting this approach. In a daily clinical practice, the decision who and when should receive the treatment is left to the treating physician, e.g., Mayo Group preference is to start the treatment when AEC is considered too high, but this threshold is not established [15]. Some authors recommend an AEC greater than 2.0 × 10^9^/L as an indication for treatment [16]. One should keep in mind that a sustained high blood HE remains frustrating both for clinician and for a patient, but it should not justify a durable CS treatment. Twelve patients with HE_us_ received steroids in our cohort. It was due to a high number of circulating blood eosinophils and the risk of organ damage. All treated patients responded to CS, and the dose was promptly reduced to maintain remission. Five patients were left on higher CS maintenance doses (10–20 mg daily), and it was due to their very high blood HE at diagnosis (from 7.9 × 10^9^ to 55.0 × 10^9^/L). In fact, the exposure to the higher CS dose was short, and therefore, the observed side effects were irrelevant. Nevertheless, all patients receive proton pump inhibitor as prophylaxis against gastric ulcers. They are also systematically monitored for potential complications. The median duration of CS treatment is 8 weeks (range 2–24 weeks). The attempts of CS discontinuation failed. A female patient who developed HES was free of CS until the cardiac failure occurred. She responded to the treatment and remains in remission with normal cardiac function while still on CS.

## Conclusions

The pathogenesis and the clinical relevance of HE_us_ remain to be elucidated. Every patient with HE_us_ requires the comprehensive evaluation to exclude various reactive and neoplastic eosinophilia-related conditions according to a contemporary consensus of multidisciplinary experts in the field of eosinophilia [3]. An international prospective study with a larger patient population and a longer follow-up is strongly required to define the risk factors of HES development. The use of CS for HE_us_ patients should be individualized paying special attention to adverse drug reactions. It seems reasonable to plan a randomized study in order to assess the benefit of CS in HE_us_.
